# Uneven distribution of HPV 16 E6 prototype and variant (L83V) oncoprotein in cervical neoplastic lesions

**DOI:** 10.1054/bjoc.2000.1247

**Published:** 2000-07-03

**Authors:** S Andersson, M Alemi, E Rylander, A Strand, B Larsson, J Sällström, E Wilander

**Affiliations:** Department of Obstetrics and Gynaecology, Danderyd hospital, Karolinska Institutet, Stockholm, Sweden; Department of Genetics and Pathology, Section of Clinical Cytology, University Hospital, Uppsala, Sweden; Department of Medical Sciences, Dermatology and Venereology, University Hospital, Uppsala, Sweden

**Keywords:** HPV16, E6 gene, polymorphism, cervical adenocarcinomas

## Abstract

A previous Swedish study revealed that both prototype and variant HPV16 E6 oncoprotein, occur in about equal numbers in high-grade cervical intraepithelial neoplasia (HCIN), whereas variant HPV16 predominates in invasive cervical squamous carcinoma. Most of the malignant HPV16 variants contain a common mutation, L83V, in the E6 oncoprotein. In the present investigation, 28 HPV16 positive, invasive cervical adenocarcinomas were collected from a total number of 131 adenocarcinomas. These HPV16-positive cases were evaluated with analysis of the E6 gene, using a recently described PCR-SSCP method for identification of the specific mutation (L83V) in the E6 gene. The results obtained were correlated to findings in 103 preinvasive, HCIN, and 31 invasive cervical squamous carcinomas also infected with HPV16. The HPV16 E6 variant L83V was present in 40% of the HCIN lesions, in 54% of the invasive adenocarcinomas, in comparison to 81% of the invasive squamous carcinomas. The difference between HCIN and squamous carcinomas was statistically significant, *P*< 0.001, whereas the difference between HCIN and invasive adenocarcinomas was not statistically significant, *P* = 0.604. Prototype HPV16 and its E6 variant L83V are both prevalent in preinvasive and invasive cervical lesions in Swedish women. However, the obvious predominance of HPV16 variant in squamous carcinomas was not seen in adenocarcinomas. A single amino-acid shift in the HPV16 E6 gene appears to result in a different transforming potential in squamous and glandular cervical lesions. © 2000 Cancer Research Campaign


British Journal of Cancer (2000) 83(3), 307–310
© 2000 Cancer Research Campaign
doi: 10.1054/ bjoc.2000.1247, available online at http://www.idealibrary.com on IDEAL®

### Uneven distribution of HPV 16 E6 prototype and variant (L83V) oncoprotein in cervical neoplastic lesions

**S Andersson[1], M Alemi[2], E Rylander[1], A Strand[3], B Larsson[1], J Sällström[2] and E Wilander[2]**

[1]Department of Obstetrics and Gynaecology, Danderyd hospital, Karolinska Institutet, Stockholm, Sweden; [2]Department of Genetics and Pathology, Section of Clinical Cytology, University Hospital, Uppsala, Sweden; [3]Department of Medical Sciences, Dermatology and Venereology, University Hospital, Uppsala, Sweden

**Summary** A previous Swedish study revealed that both prototype and variant HPV16 E6 oncoprotein, occur in about equal numbers in high-grade cervical intraepithelial neoplasia (HCIN), whereas variant HPV16 predominates in invasive cervical squamous carcinoma. Most of the malignant HPV16 variants contain a common mutation, L83V, in the E6 oncoprotein. In the present investigation, 28 HPV16 positive, invasive cervical adenocarcinomas were collected from a total number of 131 adenocarcinomas. These HPV16-positive cases were evaluated with analysis of the E6 gene, using a recently described PCR-SSCP method for identification of the specific mutation (L83V) in the E6 gene. The results obtained were correlated to findings in 103 preinvasive, HCIN, and 31 invasive cervical squamous carcinomas also infected with HPV16. The HPV16 E6 variant L83V was present in 40% of the HCIN lesions, in 54% of the invasive adenocarcinomas, in comparison to 81% of the invasive squamous carcinomas. The difference between HCIN and squamous carcinomas was statistically significant, $P < 0.001$, whereas the difference between HCIN and invasive adenocarcinomas was not statistically significant, $P = 0.604$. Prototype HPV16 and its E6 variant L83V are both prevalent in preinvasive and invasive cervical lesions in Swedish women. However, the obvious predominance of HPV16 variant in squamous carcinomas was not seen in adenocarcinomas. A single amino-acid shift in the HPV16 E6 gene appears to result in a different transforming potential in squamous and glandular cervical lesions. © 2000 Cancer Research Campaign

**Keywords**: HPV16; E6 gene, polymorphism; cervical adenocarcinomas

While analysing the E6 gene of HPV16 by DNA sequencing, it was observed that both prototype and variant HPV16 showed an almost equal prevalence in cervical intra-epithelial neoplasia III (CIN III), whereas variant HPV16 displayed a marked predominance in invasive carcinoma of Swedish women (Zehbe et al, 1998). Not all these HPV16 E6 variants were concordant, but the majority contained a common missense point mutation in base 350 T to G corresponding to a L83V shift in the E6 oncoprotein. It was therefore assumed that a point mutation in base 350 of E6 signifies progression of CIN III to invasive carcinoma.

The amino-acid 83 (L) of prototype HPV 16 E6 oncoprotein is located centrally between the two internal Zn-binding motifs, which is important for the E6 protein stability (Barbosa et al, 1989). It is surrounded by three conserved amino-acids, S-L-Y-G, identical in the genital high-risk oncogenic HPV types but differing in the benign HPV types 6 and 11 (showing AGYA and AAYA, respectively). Accordingly, the S-L/V-Y-G in E6 of genital HPV types may be a site affecting the transforming ability of the virus.

In the present investigation, a collection of invasive cervical adenocarcinomas infected with HPV16 was analysed for the presence of a point mutation in codon 83 of the E6 gene, by a novel, rapid, PCR-based method (Alemi et al, 1999). It was observed that the HPV16 E6 variant was more predominant in the invasive squamous lesions than in the adenocarcinomas.

*Received 29 November 1999*
*Revised 21 February 2000*
*Accepted 15 March 2000*

*Correspondence to:* E Wilander

### MATERIAL AND METHODS

### Tissues

From all Departments of Pathology in Sweden, histopathologically diagnosed carcinomas are collected in a national database (The Cancer Registry of the National Board of Health and Welfare in Sweden), where each specimen is given an individual topographic and diagnostic code. A total series of 131 cervical adenocarcinomas were collected from the database. Among them, 28 cases of HPV16 positive adenocarcinomas were identified. Tumours showing areas with squamous differentiation (adenosquamous carcinomas) or without obvious invasion of the stroma were excluded before the HPV testing. Further, 103 HCIN and 31 invasive cervical squamous carcinomas, with a positive HPV16 reaction, were randomly collected from the local database at the Department of Pathology in Uppsala. A subpopulation of the squamous lesions has been described before (Zehbe et al, 1998).

### HPV-testing

The HPV tests on the specimens had been performed as described earlier (Zehbe et al, 1996; Zehbe and Wilander, 1997). Briefly, the analyses were performed on extracted DNA (Lungu et al, 1992) obtained from sections of the paraffin blocks of which a preceding section had been used for morphological diagnosis. In a first sequence, the availability of tissue DNA for PCR amplification was examined by performing a β-globin test (Saiki et al, 1985). Secondly, the extracted DNA was analysed with the SHARP Signal System (Digene Diagnostics, Beltsville, MD) (Manos et al, 1989; Zehbe and Wilander, 1997) and specimens showing HPV of

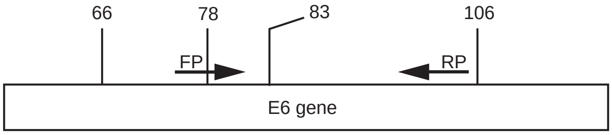

**Figure 1** Illustration of mid-portion of the HPV E6 gene. Codons 66 and 106 represent the inner Cys residue of Zn-binding domains and codon 83 the position of the mutated amino-acid (L83V). The forward primer (FP) covers a relatively rare mutation in codon 78, and reverse primer (RP)

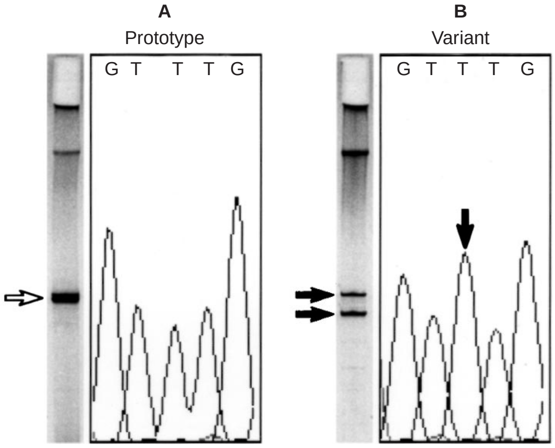

**Figure 2** PCR-SSCP (left panel) and nucleotide sequence analysis (right panel) of the E6 gene PCR products from an HPV 16. (**A**) Prototype HPV 16 displays a single SSCP band pattern, whereas variant (**B**) HPV 16 E6 produces a double (aberrant) SSCP band pattern (arrows). The sequences of prototype and variant were confirmed by non-radioactive nucleotide sequence analysis. The point mutation (vertical arrow) was found in codon 83: T to G transition causing substitution of leucine for valine high-risk type were subjected to HPV typing. HPV DNA from the L1 region was amplified with GP5+/GP6+ primers and the amplicon products were HPV-typed by single-strand conformational polymorphism (SSCP). The method has been described in detail previously (De Roda Husman et al, 1995; Zehbe et al, 1996).

The E6 gene of HPV16 positive biopsies was amplified by a primer pair constructed (DNA Technology A/S, Aarhus, Denmark) from the prototype HPV16 E6 sequence (HPV Database, Mail Stop K710, Los Alamos National Laboratory, Los Alamos, NM 87545, USA). The size of the amplicon was 176 base pairs. The PCR product was run on a SSCP gel and a polymorphism of E6 codon 83 was identified after silver staining by discrepant band pattern of the two sequence variants. The method has been described in detail before (Alemi et al, 1999). The primer pairs used for the HPV analyses are specified in Table 1.

### HPV DNA sequence analyses

In 20 cases the amplicons were recorded by DNA sequencing in order to ensure the stability of the PCR-SSCP method for visualization of the E6 codon 83 point mutation. Fluorescence-based dideoxy terminator cycle sequencing was performed using a *Taq* polymerase-based kit (Applied Biosystems Inc., Foster City, USA) according to the manufacturer's instructions and an automated DNA sequencer (Model 310, Applied Biosystems Inc.) The sequences of both sense and antisense strands of the PCR products were analysed.

### Statistical analysis

Exact two sided Pearson $\chi^2$ was utilized for the statistical analysis.

### RESULTS

### Morphology

All adenocarcinomas were of papillary or glandular type and without any squamous component. From the original collection of 131 pure adenocarcinomas, 17 were considered as in situ tumours and excluded. Of the remaining 114 tumours 71% were HPV positive mostly with type 18 (52%) and HPV16 was present in 28 (25%) of the tumours. The HPV 16 positive invasive squamous carcinomas of the cervix were of both keratinizing and non-keratinizing type.

### HPV16 E6 analysis

All HPV16 variants possessed the nucleotide 350 T to G transition corresponding to a L83V shift in the E6 oncogene. No other mutations within the amplified E6 segment were observed in any of the variant HPV16 cases using primers FP and RP (Figure 1).

In the HCIN lesions 62 of 103 cases (60%) contained the prototype sequence, whereas in the invasive squamous carcinomas only 6 of 31 (19%) tumours showed the prototype HPV16 E6.

In the invasive cervical adenocarcinomas 13 of 28 (46%) contained the prototype HPV16 E6 DNA sequence, whereas the remaining 15 (54%) tumours showed HPV16 variant E6 L83V. The strong predominance of HPV16 variant E6 seen in squamous carcinomas was not observed in the adenocarcinomas.

Women with HPV16 positive adenocarcinomas had a mean age of 47 years. The mean age of women with HPV 16 prototype E6 tumours was 49 years and for those women with HPV16 variant E6, 46 years. This difference was not statistically significant.

### HPV DNA sequence analyses

A complete correlation was observed between results obtained with the rapid PCR-SSCP method for studying E6 codon 83 polymorphism and the HPV 16 E6 DNA sequence analysis. All cases showing a double band pattern on the polyacrylamide contained a point mutation at base 350 T to G, and cases displaying a single band pattern contained a prototype HPV 16 E6 DNA sequence.

### Statistical analysis

The different distribution of prototype and variant HPV 16 E6 in HCIN and invasive squamous carcinoma of the cervix was highly

 *© 2000 Cancer Research Campaign*

**Table 1** Primer pairs used for PCR

| Primer pair | Sequence 5′–3′ | Gene position | Length (bp) | Product size (bp) |
|---|---|---|---|---|
| β-globin/PCO4 | CAACTTCATCCACGTTCACC | 54–73 | 20 | 268 |
| β-globin/GH20 | GAAGAGCCAAGGACAGGTAC | 195–176 | 20 | |
| HPV/MY09 | CGTCCMARRGGAWACTGATC[a] | L1 (HPV6) | 20 | 398 |
| HPV/MY11[b] | GCMCAGGGWCATAAYAATGG[a] | | 20 | |
| HPV/GP5+ | TTTGTTACTGTGGTAGATACTAC | L1 (HPV16) | 23 | 141 |
| HPV/GP6+ | GAAAAATAAACTGTAAATCATATTC | L1 (HPV16) | 25 | |
| HPV16 | CTAAAATTAGTGAGTATAGACATTA | E6 | 25 | 176 |
| HPV16 | CCTTATATTATGGAATCTTTGC | E6 | 22 | |

HPV, Human papillomavirus. [a]Degenerate code, M = A + C; R = A + G; W = A + T; X = G + C. [b]Biotinylated at its 5′ end.

**Table 2** Distribution of prototype and variant (L83V) HPV 16 E6 in cervical preinvasive (HCIN) lesions, and invasive squamous carcinomas and adenocarcinomas

| Histological types | HPV16 prototype E6 | HPV16 variant E6 |
|---|---|---|
| HCIN[a] | 62/103 (60%) | 41/103 (40%)[b] |
| Squamous carcinoma[a] | 6/31 (19%) | 25/31 (81%) |
| Adenocarcinoma | 13/28 (46%) | 15/28 (54%) |

[a]A subpopulation of the HCIN and squamous carcinomas has been described before (Zehbe et al., 1998). [b]The difference between HCIN and invasive carcinoma was statistically significant $P < 0.001$, whereas the difference between HCIN and invasive adenocarcinoma was not statistically significant $P = 0.604$.

significant $P < 0.001$, whereas the difference between HCIN and adenocarcinoma of the cervix was not significant $P = 0.604$.

### DISCUSSION

In a previous study, HPV16-positive HCIN and invasive cervical carcinoma were compared with respect to their DNA sequence in the transforming E6 gene. It was found that prototype HPV16 occurred in 56% of HCIN but in only 6% of the invasive carcinomas. By contrast, variant HPV was identified in 44% of HCIN and in 94% of the carcinomas. Regarding variant HPV16 E6, most of the cases displayed a common mutation T to G in nucleotide 350, corresponding to an L to V shift in amino-acid number 83 of the E6 oncoprotein (Zehbe et al, 1998). Since most HCIN lesions are considered not to progress to invasive carcinoma, the L83V mutation may signify HPV16-infected women in Sweden with a high risk of developing cervical carcinoma (Gustafsson and Adami, 1989; Östör, 1993; Moreni et al, 1995; Zehbe et al, 1998).

This suggestion is in agreement with the observation of Londesborough et al, (1996) that the proportion of different HPV 16 genotypes varied in LCIN and HCIN lesions. They found a correlation between the codon 83 mutation T to G in the HPV16 E6 gene a progression of the CIN lesion.

The amino-acid 83 of the E6 oncoprotein is surrounded by highly conserved amino-acids S-L83V-YG common in all the high-risk anogenital HPV types, but differing in the benign anogenital HPV types. For that reason it is conceivable that the SLYG and SVYG motifs denote a region with a distinct biological activity (Londesborough et al, 1996; Zehbe et al, 1998). The E6 protein of oncogenic HPV types has a transforming capacity. This property is likely to be affected by single amino-acid alterations at functional sites.

HPV 18 is considered as a virus type with a most aggressive behaviour. This proposal is mainly based on increased virus prevalence in invasive carcinoma in comparison with pre-invasive lesions. Kurman et al (1988) found HPV 18 in only 3% of CIN compared with 22% in invasive carcinoma. The codons 82–84 of E6 in prototype HPV 18 and variant HPV 16 are identical and both display an increased prevalence during progression to invasive carcinoma.

There is obvious evidence that the genital oncogenic HPV types are governed by some kind of local tropism. HPV16 has been shown to be the predominant virus type in cervical and vulvar squamous carcinomas (Hording et al, 1996; Dargent 1997; Zehbe et al, 1997; van Burden et al, 1998; Walboomers et al, 1999). HPV is present in most but not all cervical adenocarcinomas. In these tumours HPV18 is most prevalent followed by HPV 16 (Zur Hausen, 1991; Gross and Barasso, 1997) Further, our study shows the HPV 16 E6 variant L83V was considerably more predominant in the squamous carcinomas than in the adenocarcinomas. This may reflect some kind of difference in the mechanism of malignant transformation between adencarcinomas and squamous carcinomas in the cervix.

A combination of organized and opportunistic screening has reduced the incidence of squamous carcinoma substantially during the last decades, whereas the incidence of adenocarcinomas during the same time period has increased (Bergström et al, 1999) Epidemiological and molecular biological studies have shown that infection with high-risk HPV is the most important aetiological agent in the pathogenesis of cervical cancer (Bosch et al, 1995). Against this background a new screening strategy for cervical cancer has been presented in which HPV testing is combined with cytological examinations (Walboomers, 1999). Based on these conclusions it is of importance to increase our knowledge of the malignant potential of the individual HPV types and their variants and the action of various HPVs on different histological types of cervical cancer. It is possible that HPV-testing is especially important to keep back the increasing incidence of cervical adenocarcinoma.

It is emphasized, that the prevalence of oncogenic HPV types and variants, as well as the genomic background of the female population vary in different geographic areas (Bosch et al, 1995; Zehbe et al, 1998). Observations made in the Swedish female population may for that reason have a different impact in other countries (Zehbe and Tommasino, 1999).

*© 2000 Cancer Research Campaign* 

### ACKNOWLEDGEMENTS

This study was supported by the Medical Faculty of the University of Uppsala, Sweden. The technical assistance of Sonja Andersson and Cecilia Westin is appreciated. We are also grateful for the skilled photographic work of Frank Bittkowski.

### REFERENCES

Alemi M, Andersson S, Sallstrom J and Wilander E. Rapid test for identification of a human papillomavirus 16 E6 L83V variant. *Diagn Mol Pathol* 1999; **8**: 97–100.

Barbosa MS, Lowy DR and Schiller JT (1989) Papillomavirus polypeptides E6 and E7 are zinc-binding proteins. *J Virol* **63**: 1404–1407

Bergström R, Sparén P and Adami H-O. Trends in cancer of the cervix uteri in Sweden following cytologic screening. *Br J Cancer* 1999; **81**: 159–166.

Bosch FX, Manos MM, Munoz N, Sherman M, Jansen AM, Peto J, Schiffman M H, Moreno V, Kurman R and Shah KV. Prevalence of human papillomavirus in cervical cancer: a worldwide perspective. *J Natl Cancer Inst* 1995; **87**: 796–802.

Dargent D (1997) Cancer in the vulva. *Rev Prat* **47**: 1684–1689

De Roda Husman A-M, Walboomers JMM, van den Brule AJC, Meijer CJLM and Snijders PJF. The use of general primers GP5 and GP6 elongated at their 3' ends with adjacent highly conserved sequences improves human papillomavirus detection by *PCR. J Gen Virol* 1995; **76**: 1057–1062.

Gross GE and Barasso R (1997) *Human papillomavirus. A clinical atlas*. Berlin/Wiesbaden: Ullstein Mosby GmbH & Co KG

Gustafsson L and Adami HO (1989) Natural history of cervical neoplasia: consistent results obtained by an identification technique. *Br J Cancer* **60**: 132–141

Hording U, Daugaard S, Junge J and Lundvall F. Human papillomaviruses and multifocal genital neoplasia. *Int J Gynecol Pathol* 1996; **15**: 230–234.

Kurman RJ, Schiffman MH, Lancaster WD, Reid R, Jenson AB, Temple GF and Lorincz AT. Analysis of individual human papillomavirus types in cervical neoplasia: a possible role for type 18 in rapid progression. *Am J Obstet Gynecol* 1988; **159**: 293–296.

Londesborough P, Ho L, Terry G, Cuzick J, Wheeler C and Singe A. Human papillomavirus genotype as a predictor of persistence and development of high-grade lesions in women with minor cervical abnormalities. *Int J Cancer* 1996; **69**: 364–368.

Lungu O, Wright TC and Silverstein S (1992) Typing of human papillomavirus by PCR amplification with L1 consensus primer RFLP analysis. *Mol Cell Probes* **6**: 145–152

Manos MM, Ting Y, Wright DK, Leweis AJ, Broker TR and Wolinksy SM. Use of polymerase chain reaction amplification for the detection of genital human papillomavirus. *Cancer Cells* 1989; **7**: 209–214.

Moreno V, Munoz N, Bosch FX, de Sanjose S, Gonzalez LC, Tafur L, Gili M, Izarzugaza I, Navarro C, Vergara A, et al. Risk factors for progression of cervical intraepithelial neoplasm grade III to invasive cervical cancer. *Cancer Epidemiol Biomarkers Prev* 1995; **4**: 459–467.

Nuovo GJ et al (1991) Correlation of histology and detection of HPV DNA in vulvar cancer. *Gynecol Oncol* **43**: 275–280

Nuovo G, Walsh L, Gentile J, Blanco J, Koulos J and Heimann A. Correlation of the Papanicolaou smear and human papillomavirus type in women with biopsy-proven cervical squamous intraepithelial lesions. Am J Clin Pathol 1991; **96**: 544–548.

Östör AG (1993) Natural history of cervical interepithelial neoplasia: a critical review. *Int J Gynecol Pathol* **12**: 186–192

Saiki RK, Chang CA, Levenson CH, Warren TC, Boehm CD, Kazazian HH Jr and Erlich H A. Diagnosis of sickle cell anemia and beta- thalassemia with enzymatically amplified DNA and nonradioactive allele-specific oligonucleotide probes. *N Engl J Med* 1988; **319**: 537–541.

van Beurden M, ten Kate FW, Tjong-A-Hung SP, de Craen AJ, van der Vang N, Lammes FB and ter Schegget J. Human papillomavirus DNA in multicentric vulvar intraepithelial neoplasia. *Int J Gynecol Pathol* 1998; **17**: 12–16.

Walboomers JM, Jacobs MV, Manos MM, Bosch FX, Kummer JA, Shah KV, Snijders PJ, Peto J, Meijer CJ and Munoz N. Human papillomavirus is a necessary cause of invasive cervical cancer worldwide. J Pathol 1999; **189**: 12–19.

Walboomers J M. Human papilomavirus detection and cervical cancer screening. *Patologia* 1999; **32**: 270.

Zehbe I, Evander M, Edlund K, Rylander E, Wadell G and Wilander E. Nonisotopic detection and typing of human papillomaviruses (HVPs) by use of PCR-SSCP. *Diagn Mol Pathol* 1996; **5**: 206–213.

Zehbe I and Wilander E (1997) Non-isotopic ELISA-based detection of human papillomavirus-amplified DNA. *Mod Pathol* **6**: 188–191

Zehbe I and Wilander E (1997) Human papillomavirus infection and invasive cervical neoplasia: a study of prevalence and morphology. *J Pathol* **181**: 270–275

Zehbe I, Voglino G, Delius H, Wilander E and Tommasino M. Risk of cervical cancer and geographical variations of human papillomavirus 16 E6 polymorphisms. *Lancet* 1998; **352**: 1441–1442.

Zehbe I, Wilander E, Delius H and Tommasino M. Human papillomavirus 16 E6 variants are more prevalent in invasive cervical carcinoma than the prototype. *Cancer* Res 1998; **58**: 829–833.

Zur Hausen H (1991) Viruses in human cancer. *Science* 1167–1173

© 2000 Cancer Research Campaign
